# Finding the most potent compounds using active learning on molecular pairs

**DOI:** 10.3762/bjoc.20.185

**Published:** 2024-08-27

**Authors:** Zachary Fralish, Daniel Reker

**Affiliations:** 1 Department of Biomedical Engineering, Duke University, Durham, NC 27708, USA

**Keywords:** active learning, drug design, machine learning, molecular optimization, potency predictions

## Abstract

Active learning allows algorithms to steer iterative experimentation to accelerate and de-risk molecular optimizations, but actively trained models might still exhibit poor performance during early project stages where the training data is limited and model exploitation might lead to analog identification with limited scaffold diversity. Here, we present ActiveDelta, an adaptive approach that leverages paired molecular representations to predict improvements from the current best training compound to prioritize further data acquisition. We apply the ActiveDelta concept to both graph-based deep (Chemprop) and tree-based (XGBoost) models during exploitative active learning for 99 K_i_ benchmarking datasets. We show that both ActiveDelta implementations excel at identifying more potent inhibitors compared to the standard exploitative active learning implementations of Chemprop, XGBoost, and Random Forest. The ActiveDelta approach is also able to identify more chemically diverse inhibitors in terms of their Murcko scaffolds. Finally, deep models such as Chemprop trained on data selected through ActiveDelta approaches can more accurately identify inhibitors in test data created through simulated time-splits. Overall, this study highlights the large potential for molecular pairing approaches to further improve popular active learning strategies in low data regimes by enabling faster and more accurate identification of more diverse molecular hits against critical drug targets.

## Introduction

Active learning is a powerful concept in molecular machine learning that allows algorithms to guide iterative experiments to improve model performance and identify the most optimal molecular solutions [1]. Many prominent studies have shown the potential for active learning to accelerate and de-risk the identification of optimal chemical reaction conditions [2–4] and steer molecular optimization for drug discovery [5–8]. Active learning is particularly powerful during early project stages. However, one major downside is that, at these early project stages, only a very small amount of training data is available to learn from [9] which can be insufficient to support the accurate training of data-hungry machine learning models [10–11] and thereby leading to potentially sub-optimal experimental design due to an incomplete understanding of the underlying structure–activity relationship and poor calibration of predictive uncertainty. Additionally, model exploitation can lead to analog identification, which can limit the acquired knowledge and the scaffold diversity of selected hits [1].

We previously showed that leveraging pairwise molecular representations as training data can support molecular optimization by directly training on and predicting property differences between molecules [12]. Compared to classic molecular machine learning algorithms, which are trained to predict absolute property values, such paired approaches are more well-equipped to guide molecular optimization by directly learning from and predicting molecular property differences [12–15] and by cancelling systematic assay errors [12,15]. Beyond superior performance in anticipating property improvements between molecules, the molecular pairing approach shows particularly strong performance on very small datasets by benefiting from combinatorial data expansion through the pairing of molecules [12–13]. Based on these findings, we hypothesized that we could implement exploitative active learning campaigns based on a molecular pairing approach (‘ActiveDelta’) to support rapid identification of the most potent inhibitors across a wide range of benchmark drug targets.

Active learning allows algorithms to guide iterative molecular design by identifying the most valuable next experiment [1]. This can be done by selecting the compounds the model is most uncertain of to improve model performance (‘explorative’) [16–17], retrieving compounds with desired properties (‘exploitative’) [18], or a combination of both (‘balanced’) [8]. Explorative active learning provides diverse chemical structures to support model learning while exploitative approaches instead bias towards rapid identification of favorable compounds. As such, explorative strategies may not propose as many structures with desired characteristics and exploitative strategies may not add much new knowledge for the model [1]. In pursuit of quickly finding potent leads with limited data, we selected to pursue an exploitative active learning approach for this study.

**Figure 1:**
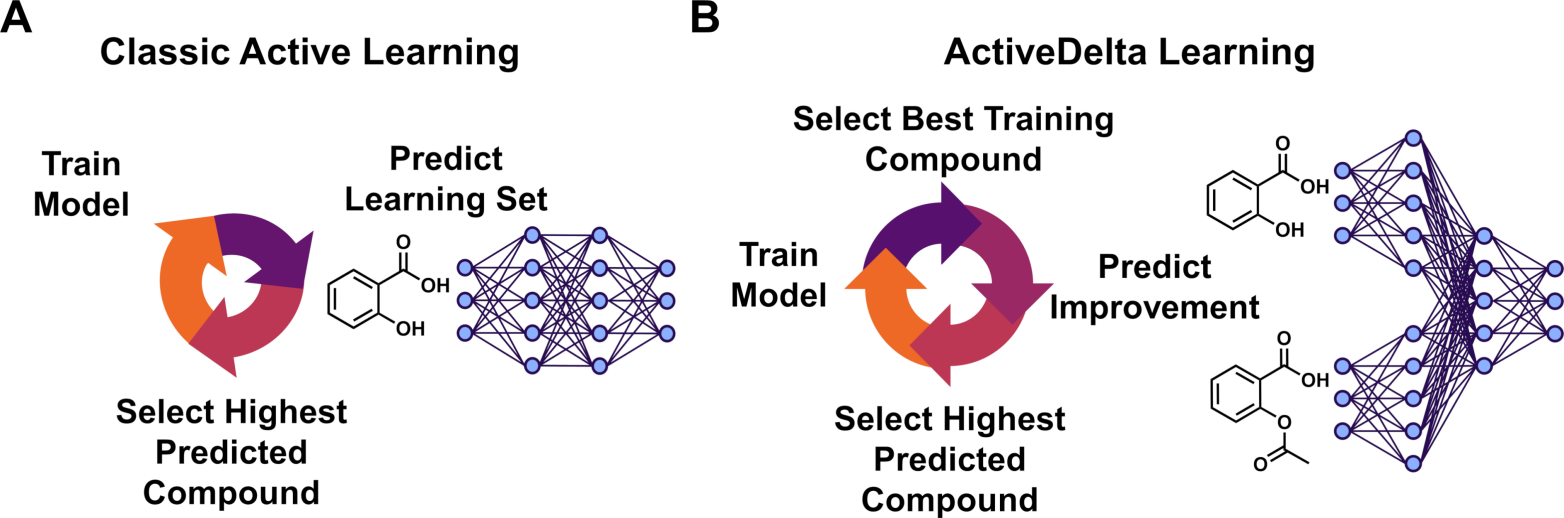
Comparison of active learning approaches. (A) Classic exploitative active learning uses individual molecular representations to predict absolute property values to select the most promising molecule from the learning set to add into the training set. (B) ActiveDelta learning uses paired molecular representations to predict molecular property improvements from the currently best training compound to select the best molecule to add to the training set that is predicted to improve the most compared to the currently best solution.

Classically during exploitative active learning, the machine learning model is trained on the available training data and the next compound to be added to the training dataset is selected based on which compound from the learning set has the highest predicted value [19] (Figure 1A). For ActiveDelta learning, training data is paired to learn property differences between molecules [12]. Then, the next compound is selected based on which compound has the greatest predicted improvement from the most promising compound currently in the training dataset (Figure 1B).

For the first time, we here present the ActiveDelta concept and evaluate the Chemprop-based [20] and XGBoost-based [21] implementations of this learning strategy against standard exploitative active learning [19] implementations of Chemprop [20], XGBoost [21], and Random Forest [22] across 99 K_i_ datasets with simulated time splits [23]. Across these benchmarks, the ActiveDelta approach quickly outcompeted standard active learning implementations, possibly by benefiting from the combinatorial expansion of data during pairing which enables the more accurate training of machine learning algorithms. The ActiveDelta implementations also enabled the discovery of more diverse molecules based on their Murcko scaffolds, possibly due to the ability to learn property differences rather than exploiting analog identification. Finally, the acquired data enabled the Chemprop algorithm to predict the most promising compounds more accurately in challenging time-split test datasets. Taken together, we believe that the ActiveDelta concept and extensions thereof hold large potential to further improve popular active learning campaigns by more directly training machine learning algorithms to guide molecular optimization and by combinatorically expanding small datasets to improve learning.

## Methods

### Datasets

Datasets were obtained from Landrum et al. [23] which utilized their simulated medicinal chemistry project data (SIMPD) algorithm to curate and split 99 ChEMBL [24] K_i_ datasets with consistent values for target id, assay organism, assay category, and BioAssay Ontology (BAO) format into training and testing sets to simulate time-based splits. Datasets were split into training and test sets at an 80:20 ratio. Duplicate molecules were removed. For initial active learning training dataset formation, two random datapoints were selected from each original training dataset and the remaining training datapoints were kept in the learning datasets (Supporting Information File 1, Figure S1). The learning dataset is the pool of molecules that models will select from during active learning [25]. Exploitative active learning was repeated three times with unique starting datapoint pairs. Test sets were not used during active learning but were used only in the test set evaluation of all algorithms.

### Model architecture and implementation

To evaluate ActiveDelta with a deep machine learning model, we used the previously established, two-molecule version of the directed Message Passing Neural Network (D-MPNN) Chemprop [20]. For our evaluation with tree-based models, we selected XGBoost [21] with readily available GPU acceleration [26]. Standard, single-molecule machine learning models were implemented using the single-molecule mode of Chemprop [12,27], XGBoost from the XGBoost library [22], and Random Forest models as implemented in scikit-learn [28]. To improve readability, we refer to our predictive pipeline consisting of our molecular pair pre-processing approach and the established two-molecule version of Chemprop as “ActiveDelta Chemprop” (AD-CP) and the standard active learning implementation of single-molecule Chemprop as “Chemprop”. Similarly, we refer to our pairing approach applied to XGBoost as “ActiveDelta XGBoost” (AD-XGB) and the standard single-molecule active learning implementation of XGBoost as “XGBoost”.

The Chemprop-based models were implemented for regression with default parameters and aggregation = ‘sum’ using the PyTorch deep learning framework. For the single-molecule Chemprop implementation, number_of_molecules = 1 while for the ActiveDelta implementation number_of_molecules = 2 to allow for processing of multiple inputs as described previously [29]. We previously optimized the number of epochs for single and paired implementations of Chemprop [12] and observed convergence of performance by 5 epochs for the paired implementation and convergence by 50 epochs for the single-molecule implementation. Based on these results, we set epochs = 5 for the ActiveDelta implementation and epochs = 50 for the single-molecule active learning implementation of Chemprop. XGBoost and Random Forest regression machine learning models were implemented with default parameters and molecules were described using radial chemical fingerprints (Morgan Fingerprint, radius 2, 2048 bits, rdkit.org) when used as inputs for these models. For the ActiveDelta implementation of XGBoost, we used default parameters and concatenated the fingerprints of each molecule in the molecular pairs to create paired molecular representations.

During active learning, standard approaches were trained on the active learning training set, consisting of two datapoints during the first iteration and increasing by 1 datapoint each subsequent iteration of active learning (Supporting Information File 1, Figure S1), and were then used to predict the absolute K_i_ value of each molecule in the learning dataset. As such, each molecule was processed individually, and predictions were made solely upon the representation of a single molecule. The datapoint with highest predicted potency was then added to the training set for the next iteration of active learning (Figure 1A). Conversely, during ActiveDelta learning, training was performed on the cross-merged training dataset to learn potency differences between molecular pairs as described previously [12]. Then, the single most potent molecule in the training set was paired with every molecule in the learning set to create new pairs for predictions on the learning data (Figure 1B). The second molecule from the molecular pair with highest predicted potency improvement was added to the training set for the next iteration of active learning, resulting in one molecule being added to the active learning training dataset at each iteration which as is commonly done in active learning except when project constraints require batch selection [1]. This datapoint would subsequently be cross-merged with all other training data compounds for ActiveDelta model retraining. For all active learning runs, analysis was repeated three times, each with a random pair of starting molecules for statistical analysis.

### Evaluation of model performance and t-SNE analysis

To measure model performance during exploitative active learning, we analyzed the models’ ability to correctly identify the compounds within the top ten percentile of most potent compounds in the learning set. For evaluations on external data, we evaluated model performance after training each model on the 100 molecules this specific model selected during exploitative active learning. The models were evaluated specifically on their ability to correctly identify the top ten percentile of the most potent compounds in the test sets and evaluations were repeated three times with three distinct initial training datasets to investigate the impact of distinct starting points.

The non-parametric Wilcoxon signed-rank test was performed for all statistical comparisons following three repeats of active learning. When presenting the number of the most potent compounds identified by each approach across 3 repeats of the 99 datasets, averages and standard deviations are presented in the text while averages and standard error of the mean are presented in the plots. For plotting of chemical space, molecules were represented by radial chemical fingerprints (Morgan Fingerprint, radius 2, 2048 bits, rdkit.org). Principal component analysis (PCA) was first performed to reduce the 2048 input dimensions to 50 dimensions before t-distributed Stochastic Neighbor Embedding (t-SNE) was applied to further reduce these 50 dimensions to 2 dimensions. PCA and t-SNE were performed with scikit-learn and plotted with matplotlib. Bar plots were created in GraphPad Prism 10.2.0. Source code and datasets used in this work can be downloaded from https://github.com/RekerLab/ActiveDelta.

## Results and Discussion

### Identifying the most potent leads using active learning on pairs

First, we evaluated how directly learning from and predicting potency differences of molecular pairs affects adaptive learning by directly comparing the performance of specific machine learning algorithms when either applied to molecular pairs or in a classic single-molecule mode. Specifically, we evaluated the ability of the D-MPNN Chemprop and the gradient boosting tree model XGBoost to adaptively learn on molecular pairs using the ActiveDelta approach compared to their standard active learning implementations in single-molecule mode (Figure 1A). As our measure of success, we analyzed all the models’ ability to identify the most potent compounds (top ten percentile) during exploitative active learning. We cold-started active learning by selecting only two random datapoints as initial training data and allowed the models to iteratively select the next molecule from the learning set that they predicted as the most potent compound to add to their training data.

**Figure 2:**
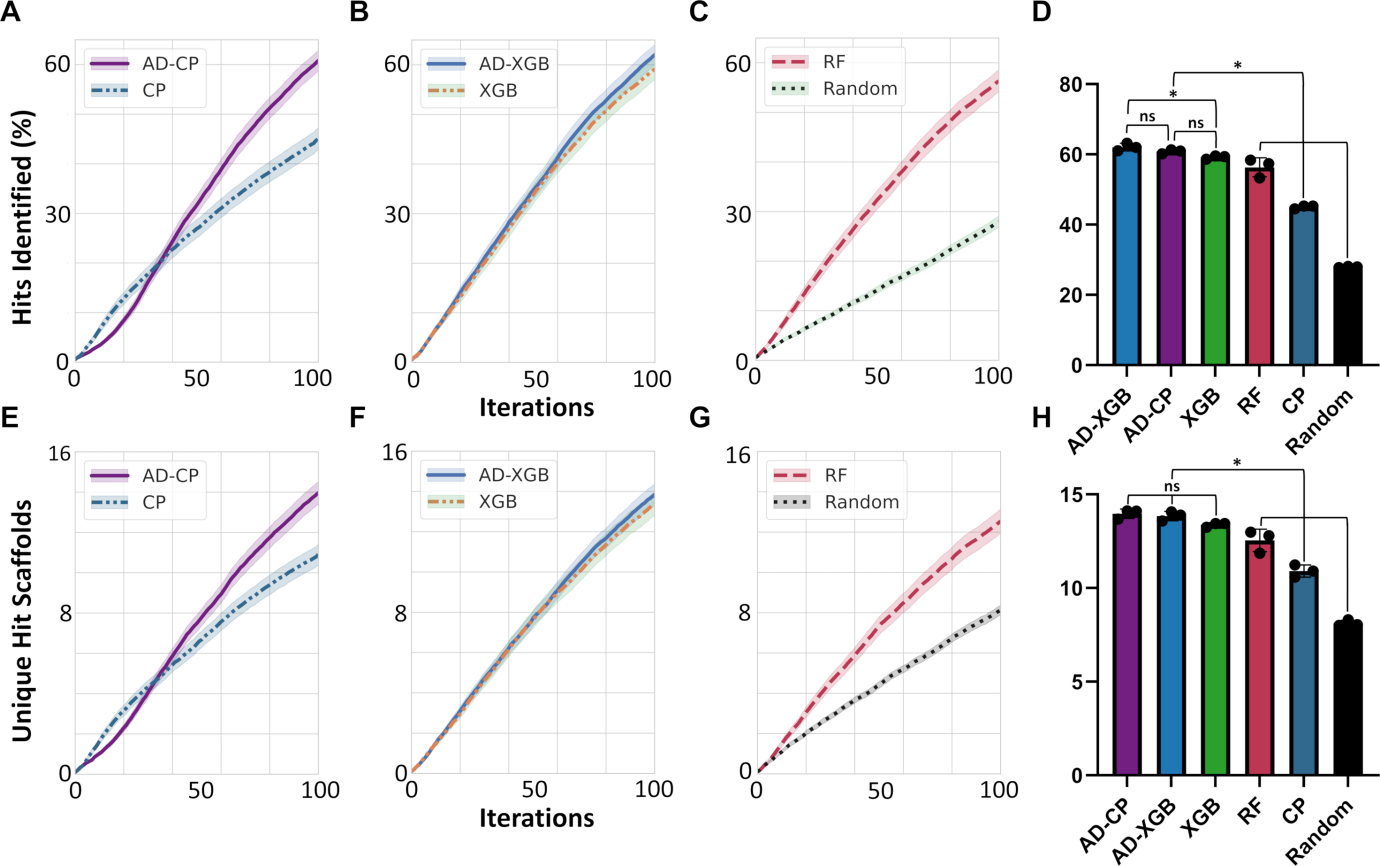
The ActiveDelta approach improves exploitative active learning performance. (A–C) The percentage of the top ten percentile most potent molecules (‘hits’) in the learning set identified over 100 iterations of active learning by (A) AD-CP and Chemprop (CP), (B) AD-XGB and XGBoost (XGB), and (C) Random Forest (RF) and random selection (Random). (D) Bar charts of the average number of identified hits of each approach at 100 iterations across all the 99 benchmarking tasks. (E–G) The number of unique scaffolds in hits selected over 100 iterations of active learning. (H) Bar charts of the average number of unique scaffolds identified by each approach at 100 iterations across all 99 benchmarking datasets. Average and standard error of the mean for three replicates across 99 K_i_ datasets after starting with two random datapoints is presented.

When comparing the deep machine learning implementations, we observed interesting patterns. AD-CP initially underperformed compared to the single-molecule implementation of Chemprop, potentially due to the increased complexity of learning and predicting potency improvements between molecular pairs compared to simply identifying analogs of the most promising compound identified so far. However, AD-CP quickly caught up and rapidly (after 35 active learning iterations) outcompeted the single-molecule active learning implementation of Chemprop. We statistically compared the performance differences of the models at 100 and 200 active learning iterations to assess their differences. We noted that AD-CP identified a statistically significantly larger fraction of the top ten percentile of most potent compounds compared to single-molecule Chemprop after 100 iterations of active learning (61% vs 45%, +6.3 leads per dataset on average, *p* = 2e − 33, Figure 2A and Supporting Information File 1, Table S1). This improved performance extended out to 200 iterations where AD-CP had identified almost 90% of the most potent inhibitors (88% vs 79%, +4.3 leads per dataset on average, *p* = 4e − 19, Supporting Information File 1, Table S1). This data overall suggests that, while the learning from and predicting of molecular pairs might be more challenging with very limited data (<35 datapoints), the pairing rapidly enables combinatorial training data expansion that allows the more effective usage of deep neural networks for the identification of the most potent compounds from limited training data until almost all hits in the learning set are selected.

A slightly different pattern emerged when comparing the tree-based implementations. AD-XGB and XGBoost initially selected similar numbers of the most potent molecules, potentially attesting to the more robust training of tree-based models on very small datasets irrespective of whether using single molecule or paired tasks. After 13 iterations, AD-XGB started consistently outperforming XGBoost. We again compared performance statistically after 100 and 200 iterations. We noted that AD-XGB was selecting a significantly larger fraction of the most potent molecules at 100 iterations (62% vs 59%, +1.0 leads per dataset on average, *p* = 0.001, Figure 2B and Supporting Information File 1, Table S1) and at 200 iterations (88% vs. 86%, +0.8 leads per dataset on average*, p* = 0.02, Supporting Information File 1, Table S1). While this difference was not nearly as stark as for the deep neural networks, the identification of an additional lead per project might still provide tangible benefits in risky real-world drug development applications where each additional lead might provide an alternative pathway to mitigate toxicities or other compound liabilities. This further attests to the power of our pairing approach and shows that tree-based machine learning models can also benefit from the pairing to identify the most potent inhibitors in adaptive learning campaigns.

When comparing the performance of the tree-based and the deep neural network-based ActiveDelta approaches, we observed that AD-CP and AD-XGB showed no statistically significant difference at 100 iterations (*p* = 0.2, Figure 2A,B, and Supporting Information File 1, Table S1) or 200 iterations (*p* = 0.7, Supporting Information File 1, Table S1). This suggests that the improved performance of the active learning campaigns is largely driven by the pairing and can be implemented with various underlying, established machine learning algorithms.

We next evaluated how the paired approaches were performing overall compared to standard, single-molecule active learning implementations. AD-CP outcompeted all standard implementations at 100 iterations (*p* < 0.002, Figure 2A–D and Supporting Information File 1, Table S1) except for XGBoost over which it showed a statistically nonsignificant improvement (*p* = 0.3, Figure 2A–D and Supporting Information File 1, Table S1) while AD-XGB outcompeted all standard implementations at 100 iterations (*p* < 0.001, Figure 2A–D and Supporting Information File 1, Table S1). By 200 iterations, both models using the ActiveDelta approach selected more of the most potent leads than any standard single-molecule active learning approach (*p* < 0.04, Supporting Information File 1, Table S1). These results highlight how a paired approach can allow models to rapidly learn in low data regimes to outcompete standard active learning implementations in identifying the most potent compounds. It also suggests that the Chemprop-based implementation requires more data than the tree-based implementation to outcompete some tree-based standard approaches, potentially hinting at the larger data requirements for deep neural networks even when combinatorially expanding datasets through pairing.

### Chemical diversity in molecular selection

Beyond their ability to identify the most potent inhibitors, we sought to determine how these approaches sampled chemical space. When analyzing the scaffold diversity of hits (i.e., the number of unique Murcko scaffolds in the set of molecules selected by the different approaches whose K_i_ values are within the top ten percentile of the most potent compounds in the complete learning set), AD-CP selected more distinct hit scaffolds than Chemprop (Figure 2E, *p* = 5e − 25 at 100 iterations) but AD-XGB’s increase in distinct hit scaffolds selected was not statistically significant compared to XGBoost (Figure 2F, *p* = 0.1 at 100 iterations). In absolute numbers (Figure 2E–H), AD-CP selected 14.0 ± 5.6 (average and standard deviation) distinct scaffolds (59.3% of all scaffolds within the hits), AD-XGB selected 13.8 ± 5.4 (59.2%), XGBoost selected 13.4 ± 5.9 (56.6%), Random Forest selected 12.5 ± 6.1 (53.1%), Chemprop selected 10.9 ± 5.2 (47.0%), and random selection selected 8.1 ± 2.4 (36.0%). AD-CP, AD-XGB, and XGBoost showed no statistically significant differences, but all three approaches outperformed all other approaches at 100 iterations.

When analyzing the scaffold diversity of all selected compounds to understand the chemical diversity of the complete training data and not just the hits, random selection had the highest scaffold diversity of all selection strategies, while AD-CP had the most diverse scaffold selection of all active learning approaches, followed by Chemprop, Random Forest, AD-XGB, and XGBoost (*p* < 0.0001 at 100 iterations, Supporting Information File 1, Figure S2). As such, AD-CP not only finds the most chemically diverse hits, with potential to create multiple lead series to enable further development of distinct scaffolds, but this approach also enriches the scaffold diversity of “negative” training data to improve future compound selection. Although the deep learning-based ActiveDelta models were not able to identify a larger number of hit compounds than the tree-based ActiveDelta implementations here, a deep learning approach appears to be more advantageous to identify more diverse hits by selecting a greater number of distinct scaffolds during exploitative active learning.

### Analyzing chemical trajectories

**Figure 3:**
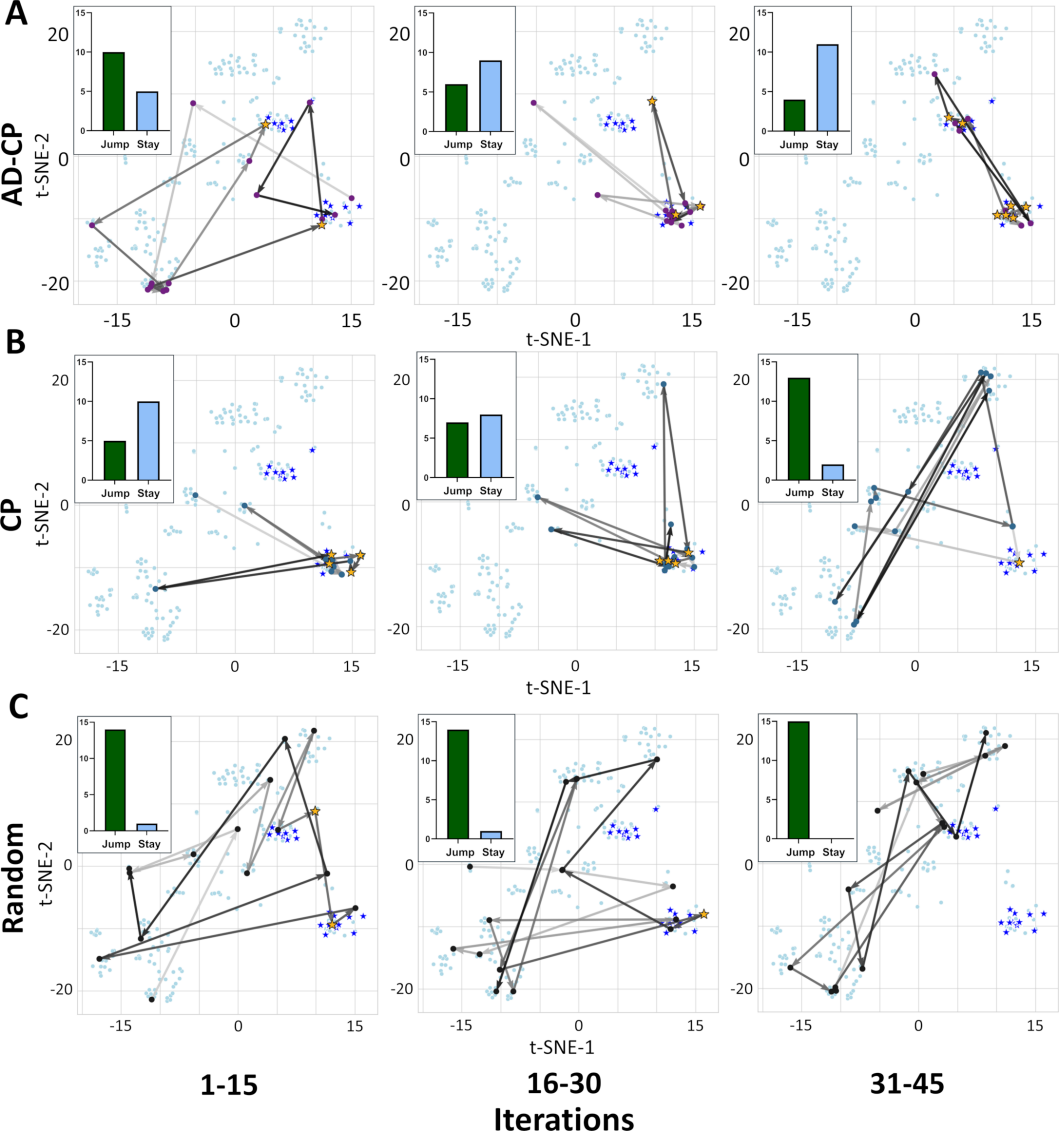
D-MPNN-based model navigation of chemical space. T-SNE of a representative dataset (CHEMBL232-1, Alpha-1b adrenergic receptor) highlighting molecules identified in the first 45 iterations for (A) AD-CP, (B) Chemprop (CP), and (C) random selection (Random). Top ten percentile most potent compounds are shown as stars and identified compounds are highlighted in yellow. The number of times a model ‘jumps’ from one cluster to another is shown in the inlet with a green bar while the times it ‘stays’ in the same cluster is shown with a light blue bar. Arrow gradient towards darker grey indicates increasing iteration number.

We next investigated how these models traversed chemical space using t-SNE analysis based on radial chemical fingerprints of molecules selected during active learning. For this analysis, we selected the most representative dataset based on similar hit retrieval rates for each algorithm on this dataset compared to the average performance of each algorithm (CHEMBL232-1, Alpha-1b adrenergic receptor). Admittedly, chemical selection trends across datasets are variable, and, as such, the following discussion is not universal but instead is a representation of the overall expected behavior of the algorithms. In the first learning iterations, AD-CP traversed chemical space broadly and jumped between clusters (Figure 3A). During 16–30 iterations, AD-CP showed a balanced behavior with equal numbers of jumps and staying within a cluster. After 30 iterations, AD-CP had identified all the relevant clusters of active compounds and largely stayed within these clusters to rapidly identify potent inhibitors. In contrast, Chemprop was more targeted at the beginning and exploited the one cluster where it could find potent inhibitors (Figure 3B). After that, Chemprop traveled more broadly and was not able to identify all clusters of potent inhibitors even after 45 iterations of learning. As expected, random selection thoroughly sampled chemical space since it is not constrained, consistently jumping between clusters (Figure 3C).

**Figure 4:**
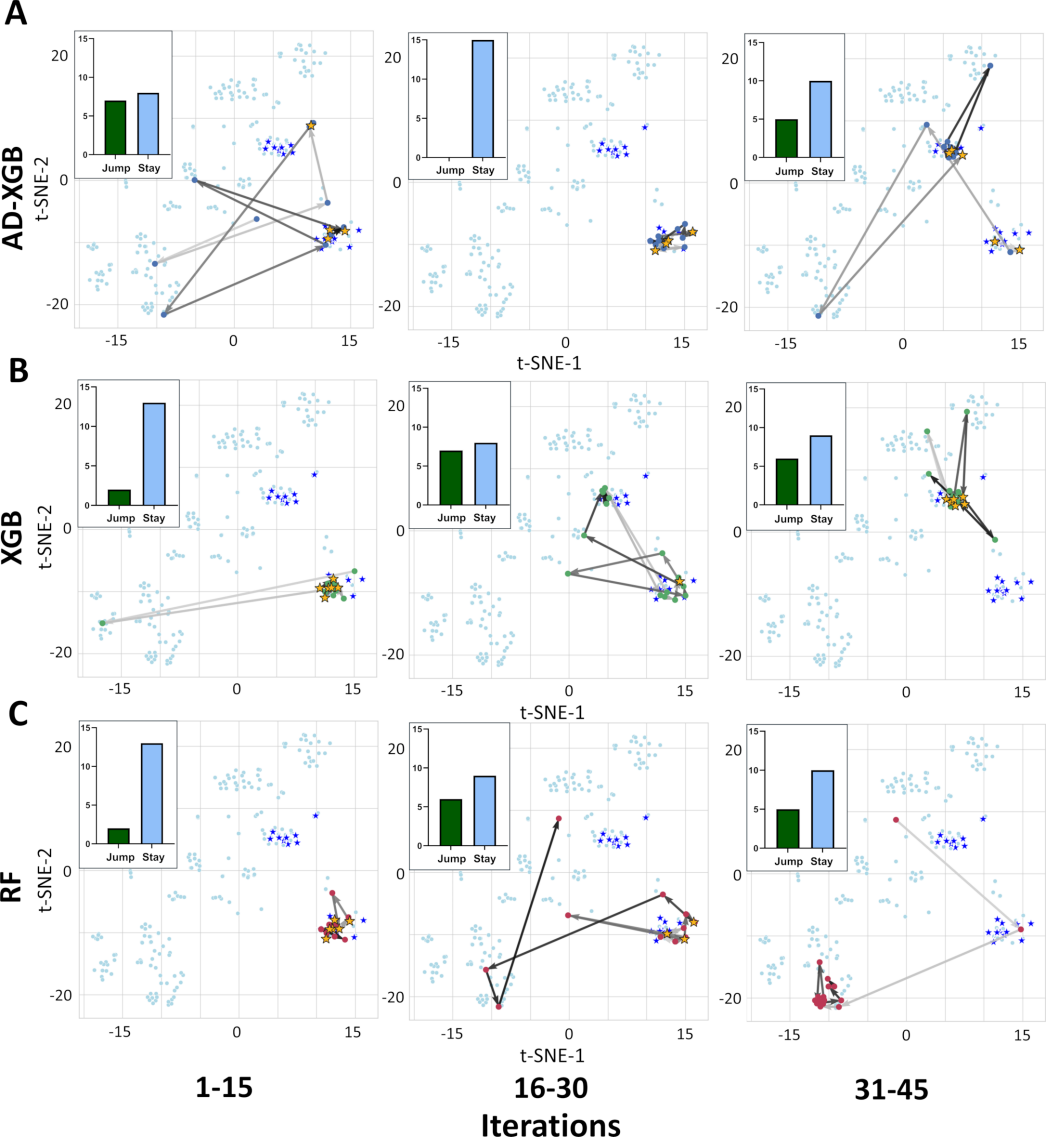
Tree-based model navigation of chemical space. T-SNE of a representative dataset (CHEMBL232-1, Alpha-1b adrenergic receptor) highlighting molecules identified in the first 45 iterations for (A) AD-XGB, (B) XGBoost (XGB), and (C) Random Forest (RF). Top ten percentile most potent compounds are shown as stars and identified compounds are highlighted in yellow. The number of times a model ‘jumps’ from one cluster to another is shown in the inlet with a green bar while the times it ‘stays’ in the same cluster is shown with a light blue bar. Arrow gradient towards darker grey indicates increasing iteration number.

Similar to AD-CP, AD-XGB exhibited broader initial search by jumping between clusters during the first learning iterations and identified a relevant cluster of potent compounds (Figure 4A). During 16–30 iterations, AD-XGB stayed within this relevant cluster until after 30 iterations where it sampled more widely again to quickly identify another relevant cluster that it stayed within to rapidly identify additional potent inhibitors. XGBoost initially showed more targeted behavior where it exploited one cluster and then broadly searched during 16–30 iterations to discover another relevant cluster (Figure 4B). Random Forest immediately exploited the one cluster where it could find potent inhibitors, but after searching more widely it did not identify any other clusters of potent inhibitors by 45 iterations of learning and instead focused on a cluster that did not contain any of the most potent molecules (Figure 4C). Altogether, these results highlight how the ActiveDelta approach can guide models to navigate diverse clusters of distinct chemistries (Figure 2E–H) by learning effectively from the initial phases of wide investigations over chemical space instead of focusing on analog identification to effectively traverse chemical space (Figure 3 and Figure 4) to identify the most potent leads (Figure 2A–D).

For an additional global analysis across all datasets instead of focused on the representative dataset, we calculated the average Tanimoto similarities of the top molecule selected by each model compared to its respective nearest neighbor in the training data using three different molecular representations (Morgan Fingerprints, MACCS Keys, and Atom Pair Fingerprints) during the initial iterations of active learning (1–15, 16–30, and 31–45) across all 99 benchmarking datasets with three repeats (Supporting Information File 1, Table S2). Random selection consistently selected the least similar molecules of all approaches (*p* < 0.005) as expected. Of all active learning approaches, AD-CP consistently selected the least similar molecules (*p* < 0.005). Conversely, Random Forest consistently selected the most similar molecules of all approaches (*p* < 0.005). AD-XGB consistently selected less similar molecules than XGBoost (*p* < 0.005) and initially selected more similar molecules than Chemprop (*p* < 0.005), but later selected less similar molecules compared to Chemprop (*p* < 0.005). Both MPNN-based models (AD-CP and Chemprop) somewhat trended towards selecting compounds with higher similarities with increasing iterations while Random Forest somewhat trended towards less similar compounds. Random selection, XGBoost, and AD-XGB exhibited no consistent trends as iterations advanced. Ultimately, AD-CP and AD-XGB consistently selected more diverse compounds than their base models (Chemprop and XGBoost, respectively, Supporting Information File 1, Table S2) while also identifying more of most potent compounds (Figure 2D) during active learning – further highlighting how the ActiveDelta approach can guide models to rapidly identify more chemically diverse hits while also collecting more diverse training data to augment model knowledge for future compound selection.

### Extrapolation to external data

Motivated by the strong ability of ActiveDelta models to effectively navigate the learning spaces, we next sought to see how readily models trained on the selected molecules by active learning could generalize to new data. We used splits that were generated to mimic real-world medicinal chemistry project data sets [23] such that the external data simulates learning from historic data to predict undiscovered “future” compounds instead of simply being selected from a separate cluster based on chemical similarity (Supporting Information File 1, Figure S4). We evaluated all the models’ performances after training on the 100 molecules they each selected from the learning set during exploitative active learning on the task of identifying novel hits (i.e., correctly predicting the top ten percentile of the most potent compounds in the test sets). Across three repeats, AD-CP correctly identified 41.3% ± 18.5 novel hit compounds in the test set on average, AD-XGB identified 40.0% ± 18.9, XGBoost identified 40.0% ± 20.4, Random Forest identified 37.9% ± 20.4, and single-molecule Chemprop identified 27.9% ± 18.7. AD-CP showed a significant improvement over Chemprop (*p* = 2e − 21), but AD-XGB showed no statistically significant difference compared to XGBoost (*p* = 0.9), possibly driven by the good performance of XGBoost alone. AD-CP was the only approach to correctly identify 100% of the hits within a test dataset while Random Forest peaked at 89%, AD-XGB and XGBoost peaked 88%, and Chemprop peaked at 83% of correctly identified hits.

In terms of chemical diversity of the novel hits identified in the test set, AD-CP identified 3.3 ± 1.7 (42.5%) of the distinct scaffolds of the novel hit compounds, XGBoost identified 3.2 ± 1.7 (41.4%), AD-XGB identified 3.1 ± 1.6 (40.6%), Random Forest identified 2.9 ± 1.7 (37.9%), and Chemprop identified 2.2 ± 1.5 (28.5%). Similar to hit identification, AD-CP showed a significant improvement over Chemprop (*p* = 8e − 24) but AD-XGB showed no statistically significant difference compared to XGBoost (*p* = 0.7). To further evaluate the ability of the algorithms to select diverse hits, we evaluated the Tanimoto similarity of their top selected hits compared to their nearest neighbors in the training data. AD-CP selected the molecules least similar to the training set (0.83 ± 0.16, *p* = 0.0003, Supporting Information File 1, Table S3), followed by Chemprop (0.85 ± 0.15, *p* = 1e − 10, Supporting Information File 1, Table S3), XGBoost (0.89 ± 0.11, *p* = 0.01, Table S3), and then Random Forest (0.90 ± 0.10, Supporting Information File 1, Table S3) and AD-XGB (0.90 ± 0.11, Supporting Information File 1, Table S3). Random Forest and AD-XGB exhibited no statistically significant difference from each other (*p* = 0.2, Supporting Information File 1, Table S3). The increased diversity in selection from the deep models, that was heightened for our paired approach, highlights how methods that allow for appropriate application of complex models in low data regimes may expand the breadth of molecular predictions based on limited knowledge. Taken together, this data suggests that the Chemprop-based AD-CP is particularly powerful at building models that can generalize to new datasets and thereby will provide medicinal chemists with options to change utilized chemistries later in the project while utilizing knowledge generated from other molecules. Its ability to identify the most diverse scaffolds in hits will also make it a most useful tool to provide medicinal chemists with various lead series for further optimization.

## Discussion

Coinciding with increased enthusiasm for machine learning methods to support drug discovery [30–31], expanded use of adaptable laboratory automation [16,32–33] will help support adaptive learning methods like active machine learning to become a cornerstone technology to guide molecular optimizations and discovery [20,34–35]. The ActiveDelta approach for active learning may efficiently guide optimization pursuits by prioritizing the most promising candidates for subsequent evaluation and could be directly integrated into robotic chemical systems to generate more potent leads through iterative design. Beyond pharmaceutical design, we expect these methods to be easily deployable for other chemical endeavors to support material design and prioritization.

Although pairwise methods like ActiveDelta exhibit increased computational costs during active learning given the combinatorial expansion of training data (Supporting Information File 1, Figure S3), these extra datapoints benefit the deep models’ abilities to learn the underlying structure–activity relationships more accurately and readily identify the most potent compounds of interest with novel scaffolds. In addition, as active learning is typically conducted for smaller datasets and in early project stages, we foresee that this combinatorial data expansion will be feasible for most active learning pipelines. Furthermore, as real-world experimentation often provides a larger bottleneck than computation, the use of more complex computational architectures with improved hit retrieval rates in place of faster, but less effective, architectures should continue to be a good choice for most real-world projects. In the future, subsampling techniques may be employed to reduce computational costs and even potentially improve performance for paired approaches. For example, it has been shown that similarity-based pairing during training compound generation for Siamese neural networks can significantly improve model efficiency [36]. Additionally, active learning-based subsampling is an autonomous and adaptive approach that has been shown to improve model performance for classification tasks [37]. As the current implementation relies on exhaustive pairing of molecules, it is optimally suited for smaller datasets but allows for data-hungry deep learning models to more adequately learn from limited data amounts. Future work should evaluate the potential of non-exhaustive pairing and subsampling strategies to allow for more efficient application of this method to larger datasets, compare against standard active learning implementations of existing methods that contrast molecules, such as Siamese neural networks [36,38–43], and apply the ActiveDelta approach to these models. Additionally, an adaptive approach that begins with an exhaustive pairing approach in low data regimes and incorporates increasing rates of subsampling as dataset size increases would be worth investigating.

Given the general notion of tree-based models’ robustness to training on smaller datasets [44], AD-CP’s ability to outcompete standard implementations of tree-based models by only 100 iterations shows particular promise for the application of deep models for low data active learning that are typically particularly troublesome for data-hungry deep learning models [9–10]. This improved performance was maintained when extrapolating to external datasets that were generated to mimic the differences between early and late compounds from true pharmaceutical optimization projects [23], indicating the generalizability of this approach.

## Conclusion

Applied to exploitative active learning, the ActiveDelta approach leverages paired molecular representations to predict molecular improvements from the best current training compound to prioritize molecules for training set expansion. Here, we have shown that this approach allows both tree-based and deep learning-based models to rapidly learn from pairwise data augmentation in low data regimes to outcompete standard active learning implementations of state-of-the-art methods in identifying the most potent compounds during exploitative active learning (Figure 2A–D) while selecting more diverse compounds (Figure 2E–H). Our t-SNE analysis suggests that ActiveDelta models will be initially forced to traverse chemical space more broadly to learn property differences between molecules rather than simply identifying analogs of promising hits (Figure 3 and Figure 4) by learning on a pairwise transformation of chemical space. The deep models using this approach also more accurately identified hits in external test sets generated through simulated temporal splits, indicating the ActiveDelta approach’s applicability and generalizability to novel chemical structures that would likely be encountered during medicinal chemistry projects. We believe that ActiveDelta and other pairwise approaches show particular promise for adaptive machine learning when training data hungry neural networks on limited data and can serve as accurate platforms to guide lead optimization and prioritization during drug development.

## Supporting Information

File 1 Supplementary figures and tables.

## Data Availability

Source code and datasets used in this work can be downloaded from https://github.com/RekerLab/ActiveDelta.
