# Supplementary material for: Finding the most potent compounds using active learning on molecular pairs
Source: Beilstein J Org Chem. 2024 Aug 27;20:2152–62. doi: 10.3762/bjoc.20.185 (PMC11368049; doi:10.3762/bjoc.20.185)
Supplement: File 1 — Supplementary figures and tables. [file Beilstein_J_Org_Chem-20-2152-s001.pdf]

BEILSTEIN JOURNAL OF ORGANIC CHEMISTRY

Supporting Information

for

Finding the most potent compounds using active learning on molecular pairs

Zachary Fralish and Daniel Reker

Beilstein J. Org. Chem. **2024,** *20,* 2152–2162. doi:10.3762/bjoc.20.185

Supplementary figures and tables

License and Terms: This is a supporting information file under the terms of the Creative Commons Attribution License (https://creativecommons.org/licenses/by/4.0). Please note that the reuse, redistribution and reproduction in particular requires that the author(s) and source are credited and that individual graphics may be subject to special legal provisions.
The license is subject to the *Beilstein Journal of Organic Chemistry* terms and conditions: (https://www.beilstein-journals.org/bjoc/terms)

Supplementary Figures

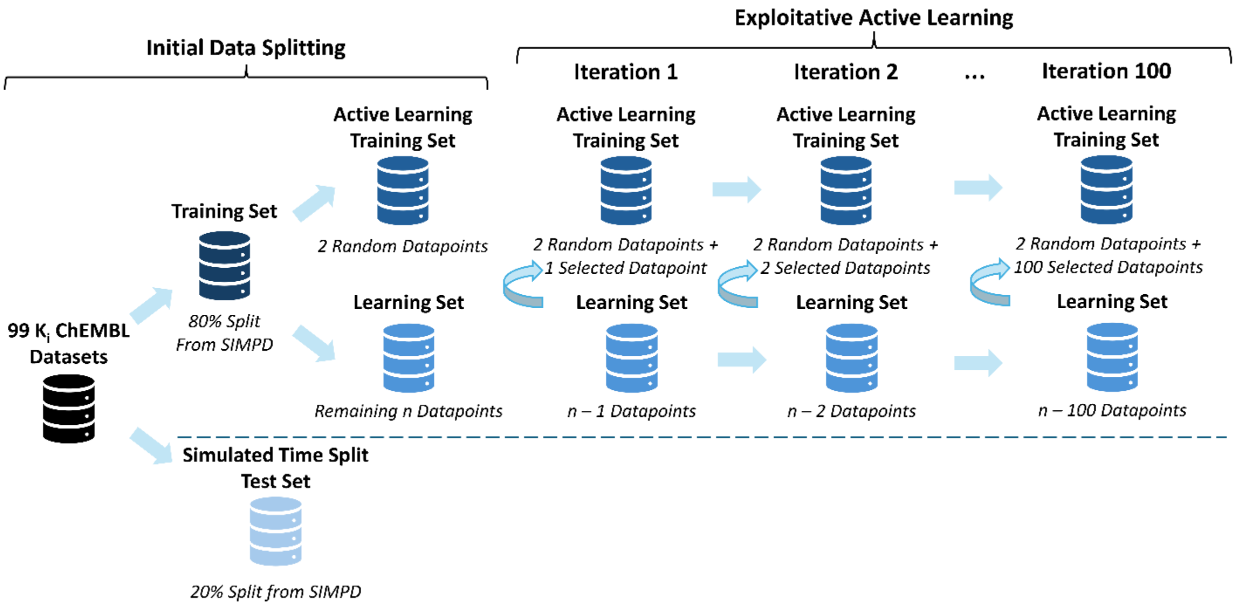

Supplementary Figure S1: Dataset splitting. Datasets were split into Training and Test Sets by the simulated medicinal chemistry project data (SIMPD) algorithm (Landrum et al. *Cheminform* 2023, 15, 119) to simulate time-based splits. Active Learning Training Sets were generated by selecting two random datapoints from the Training Sets. For ActiveDelta training, the Active Learning Training Set is then cross-merged to create all possible pairs of compounds within that set. For ActiveDelta prediction, the most potent datapoint from the Active Learning Training Set is cross-merged with the Learning Set. All other datapoints from the original Training Set were included into the initial Learning Set. During each iteration of active learning, one datapoint is selected by the model from the Learning Set to be added to the next iteration's Active Learning Training Set and removed from the next iteration's Learning Set.

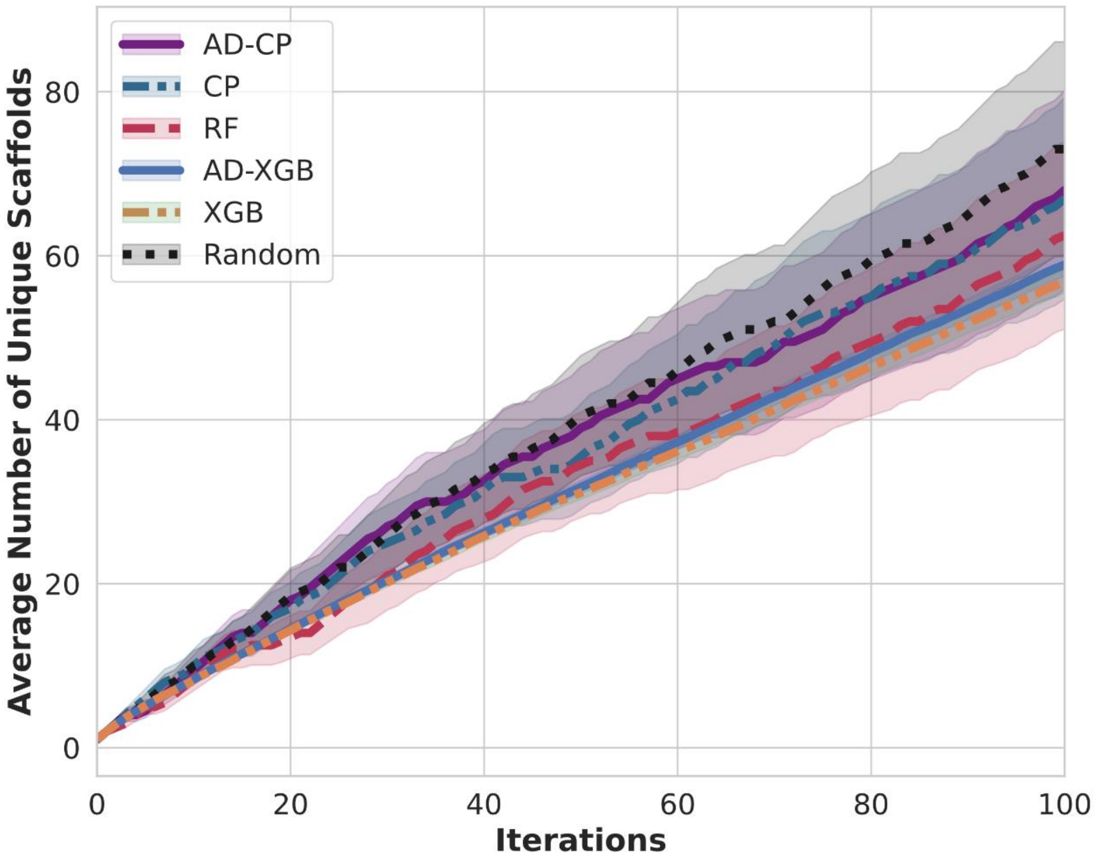

Supplementary Figure S2: Scaffold selection during exploitative active learning. The number of unique scaffolds selected by random selection (Random), Random Forest (RF), Chemprop (CP), ActiveDelta Chemprop (AD-CP), XGBoost (XGB), and ActiveDelta XGBoost (AD-XGB) is plotted over 100 iterations of active learning across 99 potency datasets after starting from two random datapoints for three repeats.

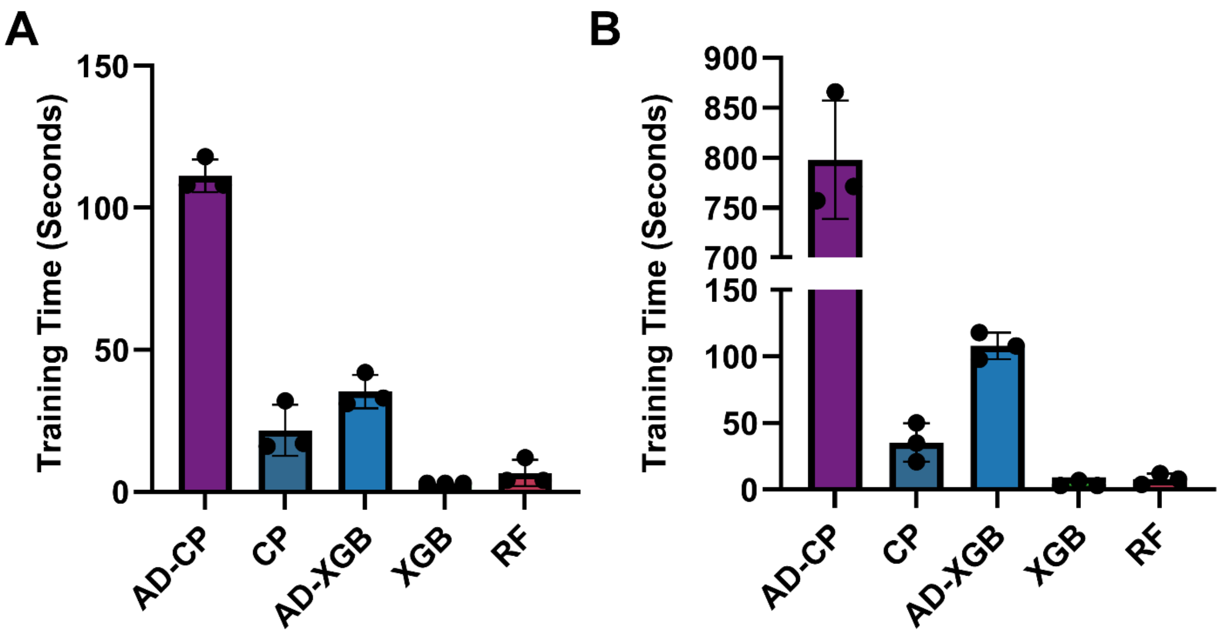

Supplementary Figure S3: Computational cost of model training. Model training time on **(A)** 100 datapoints and **(B)** 200 datapoints using an NVIDIA RTX A5000 GPU is presented in triplicates for all approaches (Random Forest (RF), Chemprop (CP), ActiveDelta Chemprop (AD-CP), XGBoost (XGB), and ActiveDelta XGBoost (AD-XGB)). Note that RF was not GPU-accelerated but was still run on the same GPU server using an Intel(R) Xeon(R) Gold 5317 CPU. At 100 training datapoints, AD-CP exhibited a 5.1-fold increase in computational cost over CP and AD-XGB exhibited a 11.8-fold increase over XGB. At 200 training datapoints, AD-CP exhibited a 22.6-fold increase over CP and AD-XGB exhibited a 24.9-fold increase over XGB.

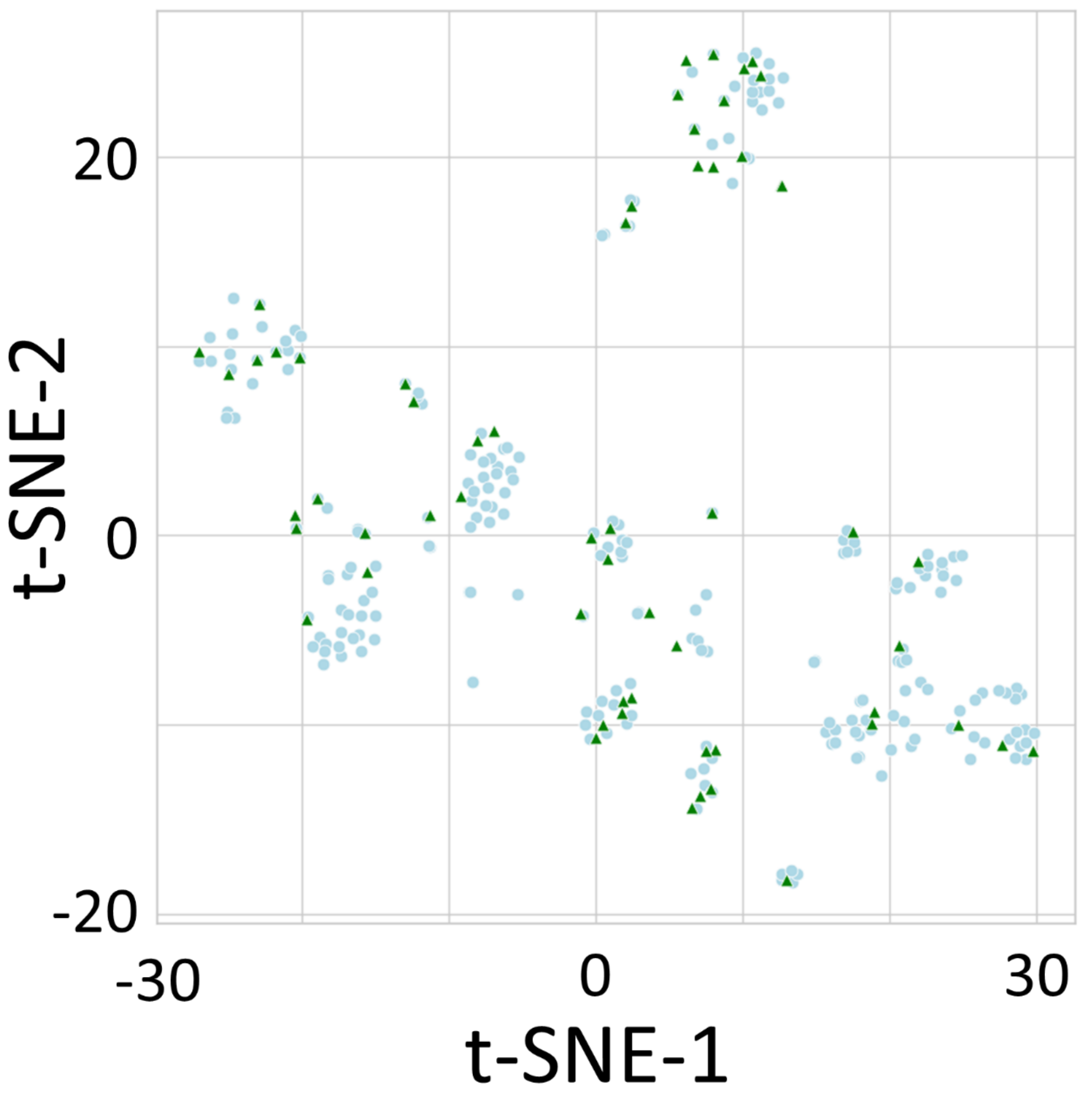

Supplementary Figure S4: T-SNE of training and test datapoints. Training datapoints are shown as blue circles and test datapoints are shown as green triangles for a representative dataset (CHEMBL232-1, Alpha-1b adrenergic receptor). Molecules were represented by radial chemical fingerprints (Morgan Fingerprint, radius 2, 2048 bits) and PCA was first performed to reduce the 2048 input dimensions to 50 dimensions before t-SNE was applied to further reduce these 50 dimensions to 2 dimensions

Supplementary Tables

Supplementary Table S1: Percent of the top ten percentile most potent leads identified by random selection (Random), Random Forest (RF), Chemprop (CP), ActiveDelta Chemprop (AD-CP), XGBoost (XGB), and ActiveDelta XGBoost (AD-XGB) during active learning across 99 K_i datasets for 200 iterations after starting from two random datapoints. Average and standard error of the mean (SEM) shown for three replicates.

Iteration	**Random**	**RF**	**CP**	**AD-CP**	**XGB**	**AD-XGB**
1	0.919 ±0.165	0.982 ±0.19	0.933 ±0.162	0.922 ±0.163	1.004 ±0.175	1.03 ±0.177
2	1.217 ±0.197	1.529 ±0.262	1.297 ±0.22	1.135 ±0.183	1.421 ±0.217	1.283 ±0.197
3	1.489 ±0.218	2.053 ±0.339	1.828 ±0.28	1.408 ±0.209	1.93 ±0.273	1.882 ±0.266
4	1.763 ±0.23	2.585 ±0.395	2.55 ±0.357	1.63 ±0.237	2.591 ±0.335	2.41 ±0.312
5	2.021 ±0.251	3.13 ±0.452	3.2 ±0.423	1.868 ±0.266	3.222 ±0.397	3.17 ±0.381
6	2.372 ±0.267	3.823 ±0.527	3.788 ±0.48	2.125 ±0.306	3.864 ±0.453	3.914 ±0.443
7	2.667 ±0.28	4.423 ±0.575	4.395 ±0.544	2.501 ±0.327	4.659 ±0.521	4.63 ±0.496
8	2.971 ±0.289	5.123 ±0.645	5.027 ±0.613	2.804 ±0.352	5.38 ±0.575	5.377 ±0.552
9	3.243 ±0.316	5.766 ±0.689	5.802 ±0.682	3.1 ±0.377	6.088 ±0.644	6.093 ±0.617
10	3.575 ±0.328	6.463 ±0.746	6.542 ±0.762	3.372 ±0.398	6.714 ±0.69	6.832 ±0.673
11	3.95 ±0.354	7.155 ±0.794	7.274 ±0.837	3.753 ±0.416	7.406 ±0.741	7.382 ±0.704
12	4.293 ±0.371	7.816 ±0.849	7.965 ±0.895	4.169 ±0.448	8.096 ±0.77	8.046 ±0.741
13	4.528 ±0.381	8.581 ±0.91	8.601 ±0.954	4.593 ±0.498	8.675 ±0.796	8.777 ±0.784

14	4.711 ±0.383	9.319 ±0.958	9.304 ±1.005	5.07 ±0.54	9.373 ±0.83	9.581 ±0.83
15	4.975 ±0.394	9.976 ±1.013	10.025 ±1.058	5.507 ±0.58	9.925 ±0.865	10.287 ±0.88
16	5.186 ±0.401	10.58 ±1.053	10.6 ±1.093	5.993 ±0.612	10.656 ±0.901	10.996 ±0.919
17	5.467 ±0.419	11.363 ±1.086	11.206 ±1.125	6.554 ±0.646	11.23 ±0.946	11.795 ±0.96
18	5.696 ±0.427	12.034 ±1.13	11.811 ±1.175	7.165 ±0.704	11.962 ±0.983	12.525 ±1.008
19	6.125 ±0.446	12.776 ±1.176	12.311 ±1.212	7.81 ±0.758	12.593 ±1.031	13.305 ±1.037
20	6.335 ±0.456	13.431 ±1.216	12.788 ±1.236	8.379 ±0.817	13.265 ±1.072	14.158 ±1.087
21	6.613 ±0.46	14.226 ±1.267	13.364 ±1.268	9.046 ±0.882	13.835 ±1.113	14.879 ±1.122
22	6.836 ±0.472	14.877 ±1.308	13.976 ±1.298	9.67 ±0.925	14.617 ±1.157	15.614 ±1.159
23	7.065 ±0.481	15.486 ±1.334	14.503 ±1.328	10.403 ±0.987	15.348 ±1.186	16.231 ±1.186
24	7.339 ±0.488	16.179 ±1.374	14.97 ±1.343	11.067 ±1.033	15.999 ±1.224	16.895 ±1.218
25	7.655 ±0.508	16.884 ±1.408	15.412 ±1.368	11.836 ±1.08	16.686 ±1.256	17.595 ±1.245
26	7.94 ±0.522	17.532 ±1.437	15.92 ±1.398	12.661 ±1.132	17.461 ±1.294	18.152 ±1.267
27	8.157 ±0.53	18.184 ±1.462	16.434 ±1.43	13.59 ±1.191	18.13 ±1.322	18.894 ±1.293
28	8.384 ±0.537	18.873 ±1.48	16.895 ±1.452	14.398 ±1.244	18.893 ±1.353	19.72 ±1.338
29	8.631 ±0.553	19.627 ±1.509	17.371 ±1.475	15.327 ±1.296	19.553 ±1.382	20.398 ±1.362
30	8.846 ±0.559	20.285 ±1.529	17.813 ±1.5	16.199 ±1.341	20.192 ±1.412	21.186 ±1.389
31	9.091 ±0.561	20.915 ±1.552	18.217 ±1.526	17.002 ±1.39	20.838 ±1.447	21.893 ±1.414
32	9.364 ±0.562	21.542 ±1.58	18.719 ±1.548	17.822 ±1.436	21.416 ±1.474	22.581 ±1.437
33	9.678 ±0.575	22.11 ±1.594	19.179 ±1.586	18.597 ±1.463	22.098 ±1.502	23.337 ±1.466
34	9.995 ±0.582	22.737 ±1.616	19.679 ±1.6	19.426 ±1.5	22.875 ±1.534	23.91 ±1.491
35	10.231 ±0.585	23.326 ±1.644	20.174 ±1.618	20.257 ±1.535	23.554 ±1.553	24.588 ±1.516

36	10.504 ±0.599	24.019 ±1.666	20.681 ±1.649	21.017 ±1.564	24.24 ±1.568	25.326 ±1.543
37	10.813 ±0.613	24.601 ±1.697	21.246 ±1.68	21.858 ±1.598	24.955 ±1.593	26.033 ±1.571
38	11.076 ±0.631	25.133 ±1.708	21.773 ±1.701	22.706 ±1.625	25.636 ±1.609	26.705 ±1.599
39	11.385 ±0.643	25.689 ±1.72	22.261 ±1.711	23.532 ±1.648	26.322 ±1.627	27.392 ±1.623
40	11.644 ±0.645	26.374 ±1.734	22.761 ±1.721	24.342 ±1.668	27.0 ±1.65	28.332 ±1.648
41	11.882 ±0.659	27.016 ±1.76	23.095 ±1.732	25.169 ±1.696	27.712 ±1.672	28.881 ±1.666
42	12.084 ±0.668	27.637 ±1.783	23.568 ±1.751	26.014 ±1.72	28.374 ±1.687	29.494 ±1.676
43	12.272 ±0.673	28.226 ±1.801	24.044 ±1.765	26.785 ±1.741	29.153 ±1.712	30.165 ±1.687
44	12.528 ±0.684	28.897 ±1.814	24.568 ±1.794	27.622 ±1.771	29.874 ±1.732	30.781 ±1.706
45	12.74 ±0.688	29.404 ±1.831	24.931 ±1.809	28.432 ±1.796	30.534 ±1.758	31.462 ±1.727
46	13.018 ±0.695	29.881 ±1.837	25.372 ±1.827	29.035 ±1.814	31.31 ±1.786	32.029 ±1.738
47	13.339 ±0.712	30.561 ±1.852	25.758 ±1.832	29.741 ±1.825	31.917 ±1.792	32.653 ±1.755
48	13.637 ±0.717	31.157 ±1.874	26.118 ±1.847	30.395 ±1.84	32.512 ±1.797	33.363 ±1.779
49	13.877 ±0.723	31.793 ±1.886	26.472 ±1.847	30.989 ±1.852	33.082 ±1.809	34.121 ±1.819
50	14.161 ±0.73	32.399 ±1.902	26.93 ±1.855	31.669 ±1.864	33.821 ±1.825	34.739 ±1.831
51	14.497 ±0.735	32.904 ±1.916	27.194 ±1.858	32.366 ±1.895	34.554 ±1.843	35.363 ±1.853
52	14.786 ±0.752	33.471 ±1.934	27.658 ±1.862	33.124 ±1.913	35.167 ±1.852	35.922 ±1.872
53	15.145 ±0.769	33.977 ±1.945	28.108 ±1.858	33.895 ±1.931	35.795 ±1.869	36.566 ±1.896
54	15.468 ±0.776	34.531 ±1.959	28.521 ±1.864	34.565 ±1.948	36.475 ±1.878	37.149 ±1.91
55	15.662 ±0.779	35.025 ±1.963	28.953 ±1.869	35.126 ±1.951	37.118 ±1.902	37.78 ±1.929
56	15.925 ±0.79	35.631 ±1.976	29.361 ±1.87	35.897 ±1.968	37.784 ±1.918	38.549 ±1.941
57	16.151 ±0.792	36.187 ±1.981	29.789 ±1.88	36.698 ±1.968	38.406 ±1.926	39.226 ±1.948

58	16.372 ±0.806	36.862 ±1.99	30.251 ±1.886	37.456 ±1.986	38.934 ±1.935	39.904 ±1.962
59	16.583 ±0.807	37.512 ±2.009	30.658 ±1.889	38.05 ±1.989	39.603 ±1.952	40.543 ±1.977
60	16.792 ±0.802	37.991 ±2.017	31.024 ±1.891	38.821 ±2.003	40.241 ±1.976	41.291 ±1.998
61	17.077 ±0.803	38.513 ±2.026	31.42 ±1.905	39.593 ±2.015	40.759 ±1.996	42.067 ±2.023
62	17.381 ±0.808	39.085 ±2.044	31.776 ±1.905	40.23 ±2.018	41.366 ±2.012	42.823 ±2.033
63	17.657 ±0.817	39.736 ±2.056	32.271 ±1.91	41.053 ±2.025	41.821 ±2.026	43.48 ±2.041
64	17.952 ±0.825	40.311 ±2.073	32.703 ±1.911	41.708 ±2.042	42.376 ±2.035	44.101 ±2.04
65	18.131 ±0.826	40.831 ±2.074	33.058 ±1.921	42.364 ±2.049	42.814 ±2.041	44.783 ±2.065
66	18.299 ±0.831	41.368 ±2.083	33.404 ±1.929	43.142 ±2.056	43.466 ±2.044	45.373 ±2.077
67	18.504 ±0.834	41.977 ±2.097	33.72 ±1.931	43.861 ±2.075	43.973 ±2.04	45.985 ±2.081
68	18.865 ±0.843	42.455 ±2.11	34.113 ±1.932	44.361 ±2.09	44.467 ±2.046	46.592 ±2.089
69	19.107 ±0.847	42.936 ±2.115	34.497 ±1.945	44.936 ±2.092	45.002 ±2.054	47.147 ±2.094
70	19.324 ±0.847	43.468 ±2.127	34.857 ±1.964	45.626 ±2.107	45.512 ±2.062	47.761 ±2.102
71	19.597 ±0.857	43.92 ±2.13	35.271 ±1.968	46.179 ±2.115	46.082 ±2.071	48.348 ±2.104
72	19.832 ±0.871	44.413 ±2.144	35.622 ±1.979	46.723 ±2.111	46.752 ±2.085	48.938 ±2.104
73	20.138 ±0.881	44.955 ±2.16	35.902 ±1.977	47.293 ±2.108	47.348 ±2.093	49.497 ±2.105
74	20.41 ±0.888	45.504 ±2.176	36.314 ±1.996	47.858 ±2.106	47.99 ±2.11	50.087 ±2.109
75	20.692 ±0.902	45.991 ±2.181	36.709 ±2.003	48.537 ±2.12	48.481 ±2.128	50.598 ±2.106
76	21.09 ±0.92	46.437 ±2.185	37.061 ±2.006	49.038 ±2.111	48.962 ±2.135	51.029 ±2.1
77	21.364 ±0.926	46.829 ±2.191	37.404 ±2.012	49.606 ±2.105	49.441 ±2.154	51.522 ±2.1
78	21.645 ±0.92	47.323 ±2.192	37.725 ±2.025	50.206 ±2.104	49.916 ±2.164	51.897 ±2.105
79	21.977 ±0.924	47.734 ±2.192	38.002 ±2.033	50.723 ±2.113	50.308 ±2.168	52.346 ±2.106

80	22.299 ±0.936	48.254 ±2.199	38.354 ±2.04	51.216 ±2.111	50.773 ±2.182	52.863 ±2.112
81	22.521 ±0.94	48.742 ±2.201	38.735 ±2.055	51.738 ±2.108	51.198 ±2.183	53.394 ±2.119
82	22.803 ±0.964	49.237 ±2.212	39.04 ±2.066	52.196 ±2.1	51.754 ±2.184	53.905 ±2.121
83	23.067 ±0.984	49.678 ±2.224	39.333 ±2.071	52.68 ±2.097	52.239 ±2.19	54.32 ±2.117
84	23.343 ±0.991	50.151 ±2.22	39.699 ±2.086	53.131 ±2.096	52.658 ±2.189	54.814 ±2.111
85	23.673 ±1.011	50.491 ±2.222	39.943 ±2.089	53.641 ±2.097	53.061 ±2.191	55.226 ±2.112
86	23.874 ±1.017	50.88 ±2.22	40.356 ±2.101	54.244 ±2.108	53.596 ±2.192	55.602 ±2.115
87	24.216 ±1.034	51.267 ±2.218	40.646 ±2.114	54.752 ±2.109	53.988 ±2.193	56.08 ±2.121
88	24.474 ±1.039	51.601 ±2.22	40.972 ±2.121	55.187 ±2.104	54.45 ±2.195	56.557 ±2.114
89	24.753 ±1.045	51.962 ±2.21	41.339 ±2.135	55.731 ±2.097	54.858 ±2.201	57.081 ±2.111
90	25.014 ±1.047	52.349 ±2.214	41.611 ±2.135	56.322 ±2.1	55.207 ±2.2	57.577 ±2.107
91	25.36 ±1.061	52.758 ±2.203	41.935 ±2.14	56.799 ±2.089	55.499 ±2.202	58.011 ±2.098
92	25.597 ±1.063	53.259 ±2.199	42.206 ±2.149	57.241 ±2.081	55.91 ±2.194	58.521 ±2.092
93	25.782 ±1.064	53.64 ±2.195	42.582 ±2.152	57.788 ±2.075	56.243 ±2.193	58.952 ±2.086
94	26.134 ±1.073	54.042 ±2.199	42.887 ±2.159	58.217 ±2.066	56.661 ±2.179	59.484 ±2.08
95	26.394 ±1.078	54.372 ±2.201	43.219 ±2.162	58.588 ±2.062	57.19 ±2.175	59.905 ±2.071
96	26.663 ±1.088	54.725 ±2.203	43.501 ±2.165	59.044 ±2.06	57.535 ±2.169	60.353 ±2.064
97	27.007 ±1.103	55.133 ±2.204	43.875 ±2.184	59.467 ±2.057	58.009 ±2.168	60.773 ±2.064
98	27.381 ±1.127	55.594 ±2.207	44.098 ±2.189	59.841 ±2.049	58.43 ±2.167	61.16 ±2.052
99	27.629 ±1.131	55.986 ±2.208	44.662 ±2.202	60.342 ±2.043	58.803 ±2.151	61.621 ±2.04
100	27.939 ±1.141	56.329 ±2.205	45.031 ±2.207	60.812 ±2.041	59.185 ±2.147	62.055 ±2.037
101	28.168 ±1.143	56.648 ±2.204	45.475 ±2.202	61.307 ±2.037	59.492 ±2.144	62.494 ±2.035

102	28.475 ±1.144	57.06 ±2.204	45.905 ±2.199	61.776 ±2.036	59.895 ±2.136	62.885 ±2.024
103	28.738 ±1.144	57.438 ±2.213	46.474 ±2.211	62.171 ±2.029	60.258 ±2.133	63.261 ±2.015
104	29.052 ±1.16	57.791 ±2.222	47.063 ±2.224	62.542 ±2.02	60.65 ±2.133	63.681 ±2.001
105	29.295 ±1.173	58.106 ±2.228	47.518 ±2.228	63.007 ±2.017	61.087 ±2.131	64.088 ±1.997
106	29.494 ±1.178	58.47 ±2.239	48.043 ±2.235	63.471 ±2.007	61.487 ±2.133	64.461 ±1.998
107	29.655 ±1.179	58.904 ±2.241	48.509 ±2.227	63.858 ±2.003	61.801 ±2.121	64.866 ±1.994
108	29.875 ±1.183	59.182 ±2.237	48.892 ±2.219	64.359 ±1.989	62.173 ±2.115	65.233 ±1.993
109	30.128 ±1.183	59.56 ±2.243	49.257 ±2.227	64.807 ±1.975	62.573 ±2.108	65.539 ±1.992
110	30.478 ±1.193	59.838 ±2.241	49.657 ±2.232	65.172 ±1.964	62.926 ±2.099	65.895 ±1.998
111	30.734 ±1.187	60.162 ±2.236	50.007 ±2.234	65.468 ±1.947	63.332 ±2.099	66.148 ±1.994
112	31.035 ±1.193	60.539 ±2.232	50.422 ±2.235	65.909 ±1.942	63.652 ±2.084	66.492 ±1.98
113	31.265 ±1.201	60.879 ±2.228	50.83 ±2.235	66.39 ±1.942	63.941 ±2.077	66.796 ±1.978
114	31.541 ±1.21	61.207 ±2.227	51.333 ±2.248	66.845 ±1.935	64.35 ±2.074	67.126 ±1.972
115	31.876 ±1.214	61.54 ±2.225	51.86 ±2.247	67.286 ±1.921	64.668 ±2.069	67.444 ±1.971
116	32.212 ±1.222	61.915 ±2.232	52.219 ±2.254	67.726 ±1.91	65.031 ±2.062	67.741 ±1.962
117	32.464 ±1.236	62.331 ±2.228	52.682 ±2.252	68.151 ±1.911	65.397 ±2.054	68.068 ±1.961
118	32.659 ±1.243	62.66 ±2.228	53.082 ±2.252	68.464 ±1.903	65.67 ±2.048	68.38 ±1.955
119	32.964 ±1.257	62.978 ±2.23	53.477 ±2.261	68.786 ±1.897	66.008 ±2.039	68.682 ±1.949
120	33.214 ±1.259	63.405 ±2.237	53.814 ±2.253	69.093 ±1.884	66.354 ±2.037	69.105 ±1.954
121	33.48 ±1.262	63.71 ±2.238	54.224 ±2.244	69.34 ±1.879	66.77 ±2.032	69.422 ±1.95
122	33.72 ±1.272	64.115 ±2.233	54.708 ±2.244	69.588 ±1.875	67.032 ±2.024	69.756 ±1.941
123	33.986 ±1.277	64.402 ±2.228	55.02 ±2.25	69.901 ±1.867	67.308 ±2.015	70.085 ±1.939

124	34.235 ±1.276	64.777 ±2.234	55.355 ±2.261	70.144 ±1.857	67.668 ±2.004	70.515 ±1.938
125	34.49 ±1.276	65.202 ±2.234	55.813 ±2.264	70.384 ±1.841	68.023 ±1.995	70.913 ±1.935
126	34.78 ±1.283	65.606 ±2.234	56.188 ±2.266	70.718 ±1.827	68.335 ±1.985	71.298 ±1.932
127	35.072 ±1.291	65.993 ±2.236	56.499 ±2.261	71.035 ±1.812	68.661 ±1.977	71.671 ±1.924
128	35.254 ±1.286	66.399 ±2.23	56.901 ±2.259	71.369 ±1.807	69.047 ±1.965	71.971 ±1.919
129	35.621 ±1.296	66.881 ±2.225	57.223 ±2.263	71.715 ±1.788	69.402 ±1.961	72.381 ±1.912
130	35.855 ±1.304	67.311 ±2.22	57.54 ±2.256	72.113 ±1.771	69.821 ±1.955	72.722 ±1.907
131	36.192 ±1.315	67.601 ±2.219	57.868 ±2.259	72.442 ±1.757	70.052 ±1.944	73.101 ±1.904
132	36.41 ±1.32	68.003 ±2.214	58.193 ±2.252	72.781 ±1.746	70.494 ±1.938	73.436 ±1.897
133	36.688 ±1.335	68.329 ±2.214	58.556 ±2.256	73.033 ±1.733	70.897 ±1.939	73.838 ±1.888
134	36.943 ±1.334	68.662 ±2.209	59.006 ±2.263	73.375 ±1.721	71.25 ±1.933	74.197 ±1.882
135	37.2 ±1.345	69.026 ±2.207	59.356 ±2.256	73.675 ±1.71	71.551 ±1.928	74.513 ±1.877
136	37.48 ±1.354	69.322 ±2.204	59.684 ±2.248	74.104 ±1.7	71.822 ±1.922	74.79 ±1.871
137	37.756 ±1.364	69.687 ±2.202	60.09 ±2.249	74.397 ±1.687	72.11 ±1.921	75.062 ±1.872
138	37.934 ±1.364	69.974 ±2.202	60.453 ±2.24	74.695 ±1.673	72.449 ±1.92	75.348 ±1.867
139	38.184 ±1.369	70.292 ±2.195	60.789 ±2.253	74.954 ±1.661	72.743 ±1.92	75.647 ±1.859
140	38.496 ±1.376	70.638 ±2.186	61.141 ±2.255	75.256 ±1.653	73.053 ±1.916	75.987 ±1.85
141	38.768 ±1.387	70.923 ±2.184	61.453 ±2.255	75.515 ±1.647	73.389 ±1.912	76.197 ±1.844
142	39.122 ±1.404	71.163 ±2.176	61.747 ±2.26	75.775 ±1.645	73.621 ±1.9	76.452 ±1.839
143	39.381 ±1.411	71.424 ±2.174	62.101 ±2.256	76.085 ±1.641	73.904 ±1.892	76.739 ±1.83
144	39.623 ±1.417	71.682 ±2.166	62.396 ±2.255	76.385 ±1.636	74.139 ±1.884	77.095 ±1.823
145	39.832 ±1.42	71.914 ±2.163	62.766 ±2.257	76.689 ±1.63	74.406 ±1.882	77.399 ±1.81

146	40.126 ±1.433	72.169 ±2.159	62.994 ±2.261	76.992 ±1.625	74.632 ±1.88	77.666 ±1.802
147	40.419 ±1.442	72.536 ±2.153	63.315 ±2.266	77.261 ±1.624	74.928 ±1.875	77.932 ±1.793
148	40.678 ±1.453	72.813 ±2.152	63.625 ±2.265	77.519 ±1.617	75.221 ±1.871	78.184 ±1.787
149	40.952 ±1.465	72.985 ±2.144	64.026 ±2.267	77.775 ±1.611	75.506 ±1.87	78.484 ±1.779
150	41.258 ±1.474	73.35 ±2.137	64.395 ±2.268	78.054 ±1.614	75.732 ±1.868	78.746 ±1.778
151	41.6 ±1.485	73.627 ±2.124	64.766 ±2.266	78.272 ±1.606	75.93 ±1.859	78.956 ±1.771
152	41.887 ±1.497	73.951 ±2.115	65.1 ±2.267	78.531 ±1.601	76.163 ±1.853	79.133 ±1.761
153	42.151 ±1.498	74.283 ±2.103	65.494 ±2.26	78.81 ±1.6	76.384 ±1.853	79.503 ±1.759
154	42.347 ±1.502	74.567 ±2.099	65.838 ±2.261	79.045 ±1.597	76.627 ±1.851	79.788 ±1.752
155	42.629 ±1.512	74.879 ±2.092	66.324 ±2.257	79.238 ±1.593	76.83 ±1.847	80.014 ±1.746
156	42.842 ±1.522	75.092 ±2.085	66.821 ±2.249	79.505 ±1.584	77.083 ±1.848	80.182 ±1.74
157	43.093 ±1.531	75.405 ±2.081	67.248 ±2.252	79.721 ±1.575	77.397 ±1.846	80.402 ±1.736
158	43.298 ±1.536	75.677 ±2.077	67.61 ±2.246	80.074 ±1.572	77.647 ±1.841	80.701 ±1.728
159	43.577 ±1.547	75.978 ±2.075	68.006 ±2.245	80.381 ±1.567	78.008 ±1.841	80.963 ±1.72
160	43.759 ±1.547	76.275 ±2.07	68.381 ±2.238	80.635 ±1.563	78.321 ±1.839	81.199 ±1.709
161	44.126 ±1.564	76.536 ±2.067	68.792 ±2.238	80.778 ±1.558	78.64 ±1.836	81.467 ±1.699
162	44.339 ±1.573	76.79 ±2.066	69.1 ±2.234	81.005 ±1.55	78.879 ±1.83	81.668 ±1.689
163	44.618 ±1.585	77.064 ±2.058	69.46 ±2.232	81.198 ±1.542	79.09 ±1.827	81.921 ±1.682
164	44.861 ±1.58	77.302 ±2.052	69.848 ±2.229	81.463 ±1.542	79.341 ±1.824	82.23 ±1.674
165	45.117 ±1.592	77.604 ±2.049	70.223 ±2.228	81.689 ±1.534	79.524 ±1.818	82.486 ±1.668
166	45.34 ±1.594	77.927 ±2.047	70.517 ±2.233	81.872 ±1.529	79.761 ±1.812	82.687 ±1.662
167	45.637 ±1.602	78.147 ±2.045	70.846 ±2.235	82.105 ±1.519	79.975 ±1.81	82.869 ±1.659

168	45.839 ±1.606	78.367 ±2.034	71.101 ±2.228	82.266 ±1.514	80.232 ±1.803	83.122 ±1.648
169	46.13 ±1.615	78.535 ±2.03	71.388 ±2.226	82.422 ±1.509	80.403 ±1.793	83.311 ±1.643
170	46.392 ±1.62	78.714 ±2.02	71.616 ±2.217	82.67 ±1.504	80.628 ±1.78	83.464 ±1.634
171	46.758 ±1.627	78.973 ±2.013	71.917 ±2.217	82.903 ±1.501	80.851 ±1.776	83.674 ±1.628
172	47.062 ±1.633	79.263 ±2.009	72.212 ±2.219	83.144 ±1.493	81.022 ±1.77	83.865 ±1.624
173	47.274 ±1.635	79.462 ±2.003	72.474 ±2.21	83.341 ±1.49	81.284 ±1.761	84.062 ±1.614
174	47.588 ±1.641	79.672 ±1.992	72.805 ±2.207	83.516 ±1.483	81.497 ±1.753	84.196 ±1.613
175	47.877 ±1.649	79.824 ±1.99	73.123 ±2.211	83.746 ±1.474	81.733 ±1.742	84.406 ±1.605
176	48.177 ±1.654	80.068 ±1.987	73.393 ±2.201	84.048 ±1.469	81.916 ±1.738	84.56 ±1.599
177	48.479 ±1.663	80.277 ±1.979	73.678 ±2.189	84.261 ±1.467	82.193 ±1.731	84.724 ±1.594
178	48.766 ±1.674	80.423 ±1.972	73.995 ±2.185	84.484 ±1.455	82.38 ±1.728	84.838 ±1.589
179	49.032 ±1.676	80.624 ±1.967	74.241 ±2.188	84.665 ±1.447	82.569 ±1.715	85.016 ±1.585
180	49.264 ±1.685	80.841 ±1.96	74.524 ±2.19	84.932 ±1.452	82.755 ±1.713	85.133 ±1.576
181	49.422 ±1.684	81.042 ±1.956	74.79 ±2.192	85.088 ±1.447	82.977 ±1.707	85.286 ±1.563
182	49.623 ±1.694	81.266 ±1.948	75.006 ±2.19	85.259 ±1.439	83.157 ±1.701	85.419 ±1.555
183	49.963 ±1.703	81.569 ±1.95	75.293 ±2.179	85.446 ±1.429	83.461 ±1.697	85.663 ±1.548
184	50.159 ±1.707	81.792 ±1.944	75.521 ±2.167	85.585 ±1.425	83.614 ±1.69	85.834 ±1.543
185	50.507 ±1.712	81.978 ±1.941	75.785 ±2.159	85.74 ±1.421	83.801 ±1.686	86.001 ±1.542
186	50.765 ±1.728	82.178 ±1.94	76.032 ±2.155	85.859 ±1.421	84.059 ±1.68	86.163 ±1.537
187	51.009 ±1.733	82.438 ±1.942	76.287 ±2.147	85.968 ±1.418	84.301 ±1.672	86.313 ±1.534
188	51.263 ±1.735	82.62 ±1.933	76.58 ±2.141	86.081 ±1.41	84.509 ±1.665	86.425 ±1.531
189	51.608 ±1.749	82.837 ±1.923	76.845 ±2.136	86.231 ±1.408	84.704 ±1.654	86.537 ±1.52

190	51.868 ±1.749	83.005 ±1.918	77.115 ±2.134	86.347 ±1.406	84.853 ±1.648	86.702 ±1.516
191	52.143 ±1.752	83.152 ±1.916	77.393 ±2.131	86.489 ±1.399	84.977 ±1.644	86.851 ±1.51
192	52.479 ±1.763	83.352 ±1.903	77.602 ±2.128	86.624 ±1.397	85.137 ±1.638	87.013 ±1.497
193	52.681 ±1.763	83.493 ±1.893	77.807 ±2.123	86.78 ±1.39	85.312 ±1.623	87.144 ±1.49
194	52.969 ±1.77	83.663 ±1.887	77.964 ±2.118	86.909 ±1.387	85.464 ±1.614	87.308 ±1.483
195	53.323 ±1.784	83.791 ±1.879	78.196 ±2.108	87.062 ±1.383	85.57 ±1.605	87.492 ±1.476
196	53.589 ±1.792	83.984 ±1.868	78.429 ±2.098	87.174 ±1.376	85.707 ±1.592	87.663 ±1.466
197	53.825 ±1.796	84.111 ±1.861	78.607 ±2.089	87.32 ±1.368	85.841 ±1.588	87.85 ±1.459
198	54.096 ±1.806	84.319 ±1.855	78.818 ±2.08	87.534 ±1.364	85.978 ±1.585	87.988 ±1.447
199	54.362 ±1.81	84.497 ±1.85	79.067 ±2.074	87.69 ±1.359	86.221 ±1.582	88.172 ±1.44
200	54.532 ±1.811	84.622 ±1.839	79.273 ±2.072	87.881 ±1.356	86.347 ±1.575	88.38 ±1.434

Supplementary Table S2: Average Tanimoto similarity of the compounds selected during active learning by random selection (Random), Random Forest (RF), Chemprop (CP), ActiveDelta Chemprop (AD-CP), XGBoost (XGB), and ActiveDelta XGBoost (AD-XGB) during the initial iterations of active learning. Three different fingerprints were used to represent the molecules, Morgan Fingerprints (radius 2, 2048 bits), MACCS Keys, and Atom Pair Fingerprints and the aggregate results are presented. Average and standard deviation are shown for three replicates.

Iterations	**Random**	**RF**	**CP**	**AD-CP**	**XGB**	**AD-XGB**
1-15	0.218± 0.066	0.458± 0.125	0.359± 0.116	0.225± 0.071	0.393± 0.124	0.367± 0.124
16-30	0.216± 0.065	0.438± 0.127	0.383± 0.120	0.308± 0.105	0.393± 0.127	0.379± 0.132

31-45	0.218± 0.068	0.433± 0.133	0.389± 0.133	0.375± 0.130	0.389± 0.132	0.371± 0.134

Supplementary Table S3: Average Tanimoto similarity of the compound selected from the external test set predicted to be most potent by Random Forest (RF), Chemprop (CP), ActiveDelta Chemprop (AD-CP), XGBoost (XGB), and ActiveDelta XGBoost (AD-XGB) compared to the respective closest compound in the training datasets. Three different fingerprints were used to represent the molecules, Morgan Fingerprint (radius 2, 2048 bits), MACCS Keys, and Atom Pair Fingerprints. "Combined" represents the aggregate results of all datapoints represented by all three fingerprints. Average and standard deviation are shown for three replicates.

Fingerprint	**RF**	**CP**	**AD-CP**	**XGB**	**AD-XGB**
Morgan	0.868 ±0.094	0.810 ±0.144	0.794 ±0.152	0.852 ±0.117	0.870 ±0.107
MACCS Keys	0.974 ±0.039	0.956 ±0.071	0.942 ±0.071	0.967 ±0.039	0.973 ±0.044
Atom Pair	0.858 ±0.105	0.792 ±0.151	0.759 ±0.184	0.850 ±0.118	0.868 ±0.111
Combined	0.900 ±0.099	0.852 ±0.147	0.831 ±0.164	0.889 ±0.114	0.904 ±0.105
